# Does Losing 5-7% of Prediabetic Body Weight from a Diabetes Prevention Program decrease Cardiovascular Risks?

**DOI:** 10.51894/001c.27627

**Published:** 2021-08-30

**Authors:** Molly Kucera, Tiffany Marchewka, Annie Craib

**Affiliations:** 1 Family Medicine Advocate Aurora Health; 2 Family Medicine Beaumont Farmington Hills

**Keywords:** prediabetes, prevention, blood pressure, lipids, glucose, obesity

## Abstract

**INTRODUCTION:**

According to the Centers for Disease Control and Prevention (CDC), one-third of adults have prediabetes (i.e., at risk for developing type 2 diabetes), a leading risk factor for cardiovascular disease. The Diabetes Prevention Program (DPP) focuses on lifestyle modifications to help participants lose 5-7% of their body weight and prevent Type 2 Diabetes. The purpose of this community-based pilot study was to investigate how successful completion of the DPP might be associated with decreases in body weight and atherosclerotic cardiovascular disease (ASCVD) risks.

**METHODS:**

Single-site, prospective cohort study. The DPP was implemented at the Farmington Village Family Practice Clinic and delivered virtually via Zoom from January 2020 through December 2020. During the first six months, participants met weekly for one hour. In the remaining six months, monthly sessions were held for one hour. Each session began with a private weigh-in followed by a uniquely designed lesson plan. A total of 14 prediabetic patients, based on hemoglobin A1c (A1c), fasting blood glucose levels, or diabetic risk calculator scores, were enrolled. For analyses, data concerning body mass index (BMI), smoking status, anti-hypertensive medications, age, race, sex, A1c, fasting blood glucose, total cholesterol, and high-density lipoprotein (HDL) levels were measured at baseline, six and 12 months. These parameters were used to calculate composite ASCVD risk percentages based on the 2013 Risk Calculator from the American Heart Association/American College of Cardiology.

**RESULTS:**

Using a series of Wilcoxon Matched Signed Rank Pair T test procedures, initial base-to six-month analyses showed a statistically significant improvement in ASCVD risk scores (p < 0.01), BMI (p < 0.01), HDL (p < 0.01), estimated weekly minutes of physical activity (p =< 0.01), and total cholesterol (p = 0.048) levels. In addition, base-to-12-month differences for ASCVD, BMI, HDL and physical activity outcomes remained statistically significant.

**DISCUSSION:**

After completion of the DPP program, both initial (base to six month) as well as follow up (base to 12-month) statistically significant improvements in ASCVD, HDL, BMI, physical activity levels, and total cholesterol were observed.

**CONCLUSIONS:**

These pilot study results are promising and consistent with the reduction of cardiovascular risk factors. These findings support the value of a structured, evidence-based educational curriculum focused on nonpharmacologic intervention to decrease weight loss and ASCVD risk scores for prediabetes adults.

## INTRODUCTION

The Diabetes Prevention Program (DPP) from the Center for Disease Control and Prevention (CDC) is a lifestyle change program designed to prevent the development of Type 2 Diabetes mellitus (T2D) in patients who have been diagnosed with prediabetes based on their hemoglobin A1c (A1C) of 5.7 to 6.4 %, fasting blood glucose of 100 to 125 mg./dl., previous diagnosis of gestational diabetes, or a screening result of high risk for T2D based on a prediabetes risk assessment test.^1,2^

The DPP is comprised of a year-long structured curriculum, broken up into two different six-month sections and providing 24 hours of instruction by a specially trained lifestyle coach to motivate and educate prediabetes patients. The first six months focus on healthy eating, increasing physical activity to a goal of at least 150 minutes per week, and developing coping mechanisms to fight challenges that can impede an individual’s success.

Coping mechanisms include how to plan when eating away from home, recognizing positive and negative food/activity cues, and teaching participants a five-step problem solving strategy that can be used to overcome barriers interfering with weight loss.^2^ The latter six months emphasize maintaining weight loss by reinforcing the skills learned, keeping patients motivated, and teaching additional techniques to overcome barriers.^2^

Participants complete a structured core curriculum in which the DPP established a goal weight loss of 5 - 7% of initial body weight during the first six months. This goal was based on data from two prior behavioral weight loss studies.^3,4^ The structured core curriculum was oriented toward how participants could primarily achieve this goal through monitoring of body weight, nutrition, weekly minutes of physical activity, and calorie restriction.

Although not all obese individuals develop diabetes, it has been well established that obesity precipitates prediabetes and development of T2D.^5^ Extra adipose tissue creates a low inflammatory state which increase proinflammatory adipokines that lead to insulin resistance and pancreatic beta-cell dysfunction. The insulin resistance and pancreatic beta-cell dysfunction worsen with sustained hyperglycemia creating a positive feedback loop and worsening organ dysfunction.^5,6^

The risk for prediabetes and diabetes increases as body mass index (BMI) increases.^7^ For this reason, the American Diabetes Association (ADA) has recommended diet and lifestyle modifications for all individuals with a BMI of 25 or higher.^7^ In 2002, the DPP Research Group conducted a 27-center randomized controlled trial with the developed curriculum and compared rates of T2D progression in participants who completed the DPP compared to those treated with pharmacotherapy alone. Results showed that DPP decreased diabetes by 58% compared to 31% in the pharmacotherapy treatment arm.^2^

Longitudinal 10 and 15-year data from the DPP Research Group continued to demonstrate that lifestyle modification group had greater success preventing the development of T2D compared to pharmacotherapy.^3,4^ Exercise and nutrition also have a known benefit to decrease blood pressure, total cholesterol, and raise high-density lipoprotein (HDL, i.e., the “good” cholesterol).^8^

The leading cause of morbidity and mortality in patients with T2D and prediabetes is atherosclerotic cardiovascular disease (ASCVD), which encompasses coronary heart disease, cerebrovascular disease, and peripheral arterial disease.^9^ Furthermore, prediabetes has a connection to ASCVD that is independent of when an individual has progressed to full diabetes.^8^

Commonly, hypertension and hyperlipidemia co-exist with prediabetes, conferring increased risks of myocardial infarction and cerebral vascular accident.^10^ One method to help determine a patient’s overall ASCVD risk is the American Heart Association (AHA) ASCVD Risk Score Calculator.^11^ This calculator considers a patient’s age, gender, race, smoking status, baseline systolic blood pressure (SBP), total cholesterol, and HDL.^11,12^ These factors are then used to calculate a composite ASCVD risk score, which provides an estimated percentage of the patient’s risk for such adverse health events during the next ten years. Those adults who score less than 5.0% are considered low risk, 5%-10% are medium risk, and greater than 10% are considered high risk, requiring statin or other lipid-lowering agents.^11^

### Purpose of Study

The purpose of this community-based pilot study was to investigate how successful completion of the DPP might be associated with decreases in BMI and ASCVD risk scores. The overall null hypothesis of the study team was that they would be unable to detect any significant changes in “matched pair” (i.e., data from same patient matched before and after DPP program) baseline-to-six-month program outcome differences observing a p value of less than 0.05 to indicate statistical significance.

## METHODS

The authors’ institutional review board had approved the project design as exempt from full review during the 2019-2020 academic year. Due to emerging COVID-19 precautions, the authors delivered the DPP program utilizing both an on-site and virtual methods from January 2020-December 2020 at Farmington Village Family Practice-East.

Inclusion criteria encompassed a diagnosis of prediabetes based on an A1c of 5.7-6.4%, fasting blood glucose of between 100-125 mg./dl., previous diagnosis of gestational diabetes, or a screening result of high risk for T2D based on providers’ prediabetes risk assessments. Exclusion criteria included age less than 18 years, current pregnancy, those already taking oral or injectable T2D medication(s), and those who were not assessed as prediabetic.

Participants completed the 16-session core DPP curriculum comprised of weekly sessions during the first six months and completed eight bimonthly maintenance sessions during the last six months. Participants were also contacted by email or phone at least once in between the maintenance sessions to help ensure program adherence.

Each hour-long program session consisted of a uniquely designed lesson plan designed by the CDC and taught by a trained lifestyle coach. Notable differences in the authors’ program compared to the original DPP program included the use of social media to enhance adherence to the program and in-person meetings needed to be converted to virtual sessions beginning in April 2020 due to the COVID-19 pandemic.

In total, fourteen sessions were held virtually. Data collection included patient body weight, A1c, total cholesterol, HDL, race, sex, age, smoking status, and SBP at baseline, six months, and twelve months. These parameters were then used to calculate the ASCVD risk score based on the American Heart Association (AHA)/American College of Cardiology (CC) 2013 Risk Calculator.^11^

An informational sheet was used by the authors to help recruit clinic patients. The CDC prediabetic risk assessment test was administered to help determine participant eligibility. Patient charts were also accessed for some data (e.g., office visit blood pressures, HDL, total cholesterol lab values levels, BMI) in compliance with HIPAA regulations. Each enrolled program participant was provided a binder containing the CDC curriculum materials and writing utensils that they were expected to bring with them to each session. When meeting virtually, patients were emailed pdf versions of weekly lessons before each session. Participants were also provided with logs to record their food intake and estimated minutes of physical activity ideally reviewed by the lifestyle coach for feedback.

The authors had received a scholarly activity support grant from the Michigan State University College of Osteopathic Medicine Statewide Campus System to help defray costs for class instructional materials and purchase scales, exercise trackers, and exercise equipment for sample patients who were unable to afford them. Other grant monies went toward purchase of healthy snacks at sessions and incentives (e.g., yoga mats) to facilitate program adherence. After program completion, those who achieved the 5-7% weight loss goal were awarded prizes with each program participant receiving an overall completion certificate for attending the majority of scheduled/rescheduled sessions.

Data were primarily analyzed using a series of Wilcoxon Matched-Pair Signed Rank Test analytic procedures using *SPSS Version 27* analytic software.^13,14^ The analyst (WC in Acknowledgements) examined the differences for each of the primary and secondary outcomes at the beginning of the DPP program to six-month data to see if there were any significant differences for each outcome observing a coefficient Alpha p value of less than 0.05 to indicate statistical significance.

## RESULTS

A total sample of N = 14 participants, mean age at time of enrollment 40.5 years (SD 8.82, range from 28 to 58 years), were enrolled in the study. The gender affiliation of the sample was 10 (71%) female, four (29%) male. Eight (57.1%) participants reporting a White racial affiliation and the remaining six (42.9%) patients some sort of Non-White affiliation. The most frequent reported level of completed education was “College Graduation” (n = eight, 57.1%) with four (28.6%) “Some College or Technical School” and two (14.3%) a “High School Graduation” level of completed education.

Measurements of A1c, SBP, HDL, total cholesterol, smoking status, and documentation of participants taking any antihypertensive medications were recorded at the first baseline session, six months, and after program completion at 12 months (Table 1).

**Table 1.** Base-to-Six-Month DPP Program Outcome Results

	Baseline (n = 14) Mean (SD)	Six Months (n = 14) Mean (SD)	12 months (n = 14) Mean (SD)	Significance of Base-to-Six-Month P-value *
Composite ASCVD Score	**2.00 (SD 1.26)**	**1.26 (1.02)**	**1.08 (0.89)**	**< 0.01**
Hemoglobin A1c	**5.65 (0.35)**	**5.55 (0.27)**	**5.48 (0.29)**	**0.110**
Total Cholesterol	**190.64 (27.49)**	**178.71 (20.99)**	**175.14 (17.04)**	0.048
HDL	**40.86 (11.09)**	**48.00 (13.46)**	**48.00 (13.46)**	**< 0.01**
Body Mass Index	**40.10 (10.10)**	**36.89 (9.14)**	**36.89 (9.14)**	**< 0.01**
Estimated Minutes of Weekly Physical Activity **	**115.83 (150.11)**	**350.12 (150.11)**	**406.78 (322.46)**	**< 0.01**

SD = standard deviation

* Wilcoxon Matched Pair Signed Rank T Tests<br>** measured during “early” (i.e., first three), “middle’ (i.e., middle three), and”later” (i.e., final three) sessions.

### Outcome Differences

**Figure 1.**
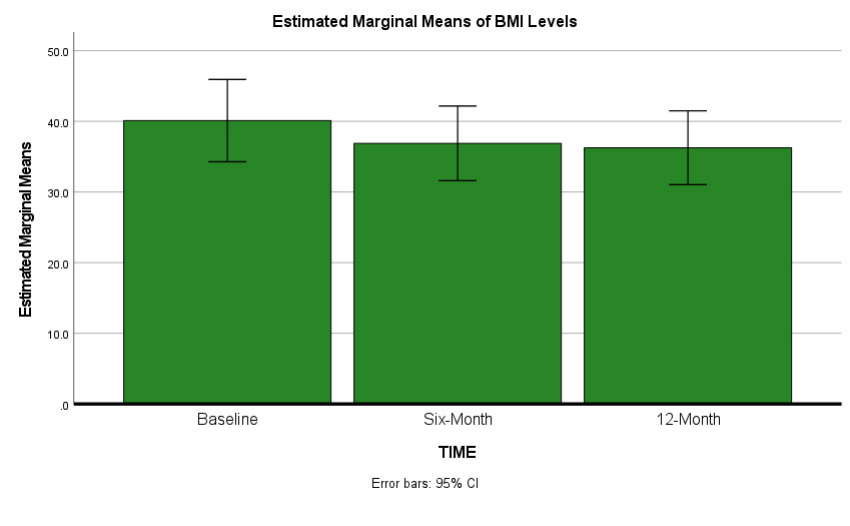
Estimated Marginal Means BMI Levels

**Figure 2.**
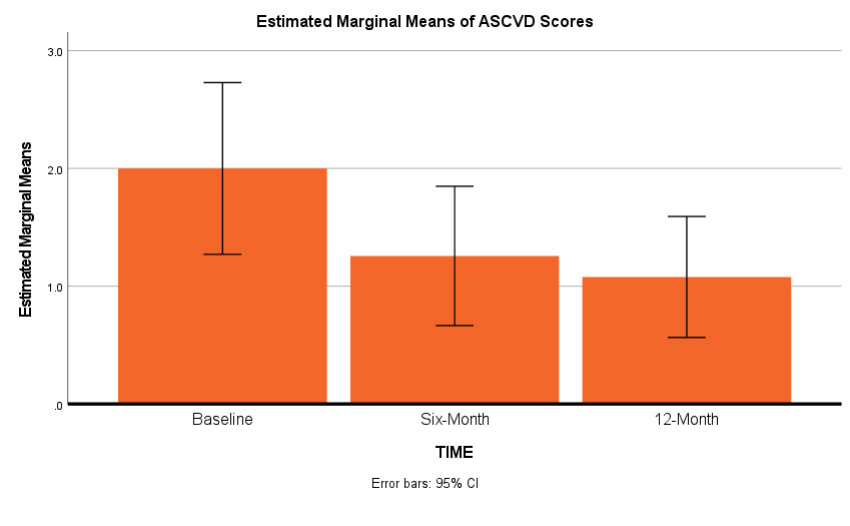
Estimated Marginal Means ASCVD Risk Scores

As depicted in Table 1 and Figures 1 and 2, both mean BMI (p < 0.01) and mean ASCVD risk scores (p < 0.01) significantly decreased from baseline to six-month, (although less significantly from six to 12 months). NOTE: Base-to-12-month p values for four selected outcomes also remained statistically significant: ASCVD scores (p < 0.01), HDL p < 0.01, BMI levels p < 0.01, minutes of physical activity (p < 0.01).

## DISCUSSION

In this community-based pilot study, statistically significant improvements were demonstrated for ASCVD risk scores, BMI levels and several other outcomes upon the completion of the DPP. These results suggest that the value of a structured, evidence-based educational curriculum focused on non-pharmacologic strategies can decrease cardiovascular risks for patients at risk for developing T2D.

The DPP^1,2^ and Kokkinos, et al.^15^ showed similar findings for BMI, HDL and reported physical activity levels. These kinds of healthy changes highlight the importance of exercise and the need to motivate patients to increase their physical activity to delay or even prevent them from starting statin or other cholesterol lowering medications and serious side effects such as statin-induced myopathy, hepatotoxicity, pancreatitis, and Stevens-Johnson Syndrome.^16^

In our study, a statistically significant mean BMI decrease throughout the 12 -month study window was observed, with the greatest difference noted in the first six months of the program (even though overall sample weight losses were apparently maintained). This is consistent with the intentions of the DPP, as the goal was to lose 5-7% body weight during the first six months and then maintain weight for the remaining of the program.

In 2001, Anderson et. al analyzed a total of 9,536 patients and demonstrated that a 1% increase over desirable BMI could decrease the risk of cardiovascular disease by 3.3% in females and 3.6% in males.^17^ These type of weight decreases have been shown to help decrease blood pressure levels as well as improve total cholesterol, LDL, and HDL lab values.^8,12,17^ For example, Kawamoto, et al. showed decreased central blood pressure in elderly Japanese individuals with modest weight loss after completion of their 12-week exercise program.^18^

In our sample, A1c levels decreased minimally, representing the complexity of studying changes of Type 2 Diabetes patients remain. However, Umpierre et. al performed a 2011 meta-analysis featuring 47 randomized controlled trials encompassing 8,538 patients that showed a combined structured exercise program of at least 12 weeks and dietary changes contributed to a statistically significant decrease in A1c levels.^19^

Reductions in total cholesterol reduction just reached statistical significance from base to six months (p = 0.048) but fell out of significance at 12 months. Similarly, Savolainen, et al. noted no statistically significant changes in total cholesterol with weight loss from lifestyle modifications.^20^ Further larger-sample studies are certainly needed to verify a statistically significant change in total cholesterol.

### Study Limitations

Several limitations to our study design included a small sample size, lack of ethnic diversity, possible self-selection bias (i.e., more motivated patients were willing to enroll) and a possible “preferred response” bias to inflated weekly minutes of reported physical activity. This study should be repeated on a larger, more ethnically diverse population to confirm these results.

## CONCLUSION

In conclusion, participation in a structured, evidence-based educational curriculum like the DPP has the potential to significantly decrease a prediabetes patient’s ASCVD risks, lab values, and tendency to develop T2D. Future larger-sample replication studies with prediabetes patients and those already diagnosed with T2D to see if a structured lifestyle modification programs can more greatly impact certain subgroups of patients’ ASCVD risks are needed.

### Conflict of Interest

None
